# A cAMP and Ca^2+^ coincidence detector in support of Ca^2+^-induced Ca^2+^ release in mouse pancreatic β cells

**DOI:** 10.1113/jphysiol.2005.087510

**Published:** 2005-04-28

**Authors:** Guoxin Kang, Oleg G Chepurny, Michael J Rindler, Leon Collis, Zina Chepurny, Wen-hong Li, Mark Harbeck, Michael W Roe, George G Holz

**Affiliations:** 1 Department of Physiology and Neuroscience, New York University School of MedicineNew York, NY 10016, USA; 2 Department of Cell Biology, New York University School of MedicineNew York, NY 10016, USA; 3 Department of Cardiology, New York University School of MedicineNew York, NY 10016, USA; 4 Departments of Cell Biology and of Biochemistry, University of Texas Southwestern Medical Center5323 Harry Hines Blvd, Dallas, TX 75390-9039, USA; 5 Department of Medicine, Mc1027, amb m172, University of Chicago5841 South Maryland Avenue, Chicago, IL 60637

## Abstract

The blood glucose-lowering hormone glucagon-like peptide-1 (GLP-1) stimulates cAMP production, promotes Ca^2+^ influx, and mobilizes an intracellular source of Ca^2+^ in pancreatic β cells. Here we provide evidence that these actions of GLP-1 are functionally related: they reflect a process of Ca^2+^-induced Ca^2+^ release (CICR) that requires activation of protein kinase A (PKA) and the Epac family of cAMP-regulated guanine nucleotide exchange factors (cAMPGEFs). In rat insulin-secreting INS-1 cells or mouse β cells loaded with caged Ca^2+^ (NP-EGTA), a GLP-1 receptor agonist (exendin-4) is demonstrated to sensitize intracellular Ca^2+^ release channels to stimulatory effects of cytosolic Ca^2+^, thereby allowing CICR to be generated by the uncaging of Ca^2+^ (UV flash photolysis). This sensitizing action of exendin-4 is diminished by an inhibitor of PKA (H-89) or by overexpression of dominant negative Epac. It is reproduced by cell-permeant cAMP analogues that activate PKA (6-Bnz-cAMP) or Epac (8-pCPT-2′-*O*-Me-cAMP) selectively. Depletion of Ca^2+^ stores with thapsigargin abolishes CICR, while inhibitors of Ca^2+^ release channels (ryanodine and heparin) attenuate CICR in an additive manner. Because the uncaging of Ca^2+^ fails to stimulate CICR in the absence of cAMP-elevating agents, it is concluded that there exists in β cells a process of second messenger coincidence detection, whereby intracellular Ca^2+^ release channels (ryanodine receptors, inositol 1,4,5-trisphosphate (IP_3_) receptors) monitor a simultaneous increase of cAMP and Ca^2+^ concentrations. We propose that second messenger coincidence detection of this type may explain how GLP-1 interacts with β cell glucose metabolism to stimulate insulin secretion.

Pancreatic β cells express GTP-binding protein-coupled receptors that mediate stimulatory actions of the blood glucose-lowering hormone glucagon-like peptide-1-(7–36)-amide (GLP-1) on insulin biosynthesis and secretion (Kieffer & Habener, 1999). Insulinotropic actions of GLP-1 are mimicked by exendin-4 (Ex-4), a peptide related in structure to GLP-1. Ex-4 acts as a high-affinity agonist at the GLP-1 receptor (GLP-1-R) and, as is the case for GLP-1, it is currently under investigation for use in the treatment of diabetes mellitus (Holz & Chepurny, 2003). Activation of the GLP-1-R on β cells initiates a complex series of signalling events that include cAMP production, membrane depolarization, an increase of intracellular calcium concentration ([Ca^2+^]_i_), and exocytosis (Thorens, 1992; Holz *et al.* 1993, 1995, 1999; Gromada *et al.* 1995, 1998*a*,*b*; Bode *et al.* 1999; Nakazaki *et al.* 2002; Eliasson *et al.* 2003). Because multiple processes within the β cell are regulated in a cAMP- and Ca^2+^-dependent manner, it is of interest to ascertain how these two second messengers interact. In particular, the potential importance of second messenger coincidence detection as a determinant of GLP-1-R signal transduction has yet to be explored fully (Holz & Habener, 1992).

Coincidence detection in biological systems is a phenomenon in which two or more complementary signals interact synergistically to generate a cellular response. Neither signal is an adequate stimulus in the absence of its complement. The type VIII isoform of adenylyl cyclase expressed in β cells acts as a molecular coincidence detector because it is stimulated not only by G_S_ GTP-binding proteins, but also by Ca^2+^/calmodulin (Pipeleers *et al.* 1985; Schuit & Pipeleers, 1985; Delmeire *et al.* 2003). Coincidence detection also exists when the activity of an effector molecule is governed by multiple second messengers. This may be the case for intracellular Ca^2+^ release channels (ryanodine receptors, RYR; inositol 1,4,5-trisphosphate (IP_3_) receptors, IP_3_-R), the opening of which is reported to be facilitated by Ca^2+^ and cAMP (Marx *et al.* 2000; Bruce *et al.* 2003). Because GLP-1 stimulates cAMP production, and because β cell glucose metabolism stimulates influx of Ca^2+^ through voltage-dependent Ca^2+^ channels (VDCCs), it is predicted that GLP-1 and glucose should interact synergistically to gate Ca^2+^ release channels from a closed to open state. Indeed, second messenger coincidence detection of this type might explain the unusual interaction of GLP-1 and glucose to mobilize an intracellular source of Ca^2+^ in the β cell (Gromada *et al.* 1995; Bode *et al.* 1999; Holz *et al.* 1999; Kang *et al.* 2001, 2003; Kang & Holz, 2003; Sasaki *et al.* 2002; Tsuboi *et al.* 2003; Dyachok & Gylfe, 2004).

Here we demonstrate that Ex-4 acts via cAMP, protein kinase A (PKA), and the Epac family of cAMP-regulated guanine nucleotide exchange factors (cAMPGEFs; also known as Epac1 and Epac2; Holz, 2004*a*) to sensitize Ca^2+^-induced Ca^2+^ release (CICR) mediated by the RYR and IP_3_-R. Sensitization allows CICR to be triggered by the uncaging of Ca^2+^ in INS-1 cells or mouse β cells loaded with a photolabile Ca^2+^ chelator (NP-EGTA; Ellis-Davies *et al.* 1994). Because the uncaging of Ca^2+^ fails to stimulate CICR in the absence of cAMP-elevating agents, it is concluded that there exists in β cells a process of second messenger coincidence detection whereby intracellular Ca^2+^ release channels monitor a simultaneous increase of cAMP and Ca^2+^ concentrations. Some of these findings have been published in preliminary form (Kang *et al.* 2005).

## Methods

### Islet isolation and cell culture

Islets were isolated from male C57BL/6 mice fed *ad libitum* (20–25 g body weight; Charles River Laboratories, Inc., Wilmington, MA, USA). The mice were anaesthetized by inhalation of CO_2_ (100%; 2–3 min exposure), and were killed by cervical dislocation. Surgical procedures for removal of the pancreas were performed in accordance with NYU School of Medicine policies governing the ethical use of mice for experimentation (IACUC Protocol no. 040602-01). After digestion of the pancreas with collagenase P (Roche Applied Science, Indianapolis, IN, USA; 2 mg ml^−1^ dissolved in RPMI 1640 medium), batches of 150–200 islets were subjected to mild trypsinization and were dispersed by trituration in a Ca^2+^-free saline in order to generate a single cell suspension. Isolated cells were then allowed to adhere to glass coverslips (25CIR-1; Fisher Sci.) coated with concanavalin A (type V; Sigma-Aldrich, St Louis, MO, USA). Primary cultures were maintained in a humidified incubator (95% air, 5% CO_2_) at 37°C in RPMI 1640 supplemented with 10% FBS, 100 units ml^−1^ penicillin G, and 100 μg ml^−1^ streptomycin. β cells were identified on the basis of their large diameter and granular appearance. INS-1 cells (passage numbers 70–90) were maintained in RPMI 1640 containing 10 mm Hepes, 11.1 mm glucose, 10% FBS, 100 units ml^−1^ penicillin G, 100 μg ml^−1^ streptomycin, 2.0 mml-glutamine, 1.0 mm sodium pyruvate, and 50 μm 2-mercaptoethanol (Asfari *et al.* 1992). INS-1 cells were passaged by trypsinization and subcultured once a week. All reagents for cell culture were obtained from Invitrogen-Life Technologies (Rockville, MD).

### Measurement of [Ca^2+^]_i_

The fura−2 loading solution consisted of standard extracellular saline (SES) containing (mm): 138 NaCl, 5.6 KCl, 2.6 CaCl_2_, 1.2 MgCl_2_, 10 Hepes, 11.1 d–glucose and supplemented with 1 μm fura−2 AM (Molecular Probes Inc., Eugene, OR, USA), 2% FBS, and 0.02% Pluronic F-127 (w/v; Molecular Probes Inc.). Cells were exposed to the fura−2 loading solution for 20–30 min at 22°C. Experiments were performed in SES at 32°C using a TE300 inverted microscope (Nikon, Melville, NY, USA) equipped with a temperature-controlled stage (Medical Systems Corp., Greenvale, NY, USA) and a 100× Nikon UVF oil immersion objective (NA 1.3). Microfluorimetry was performed ratiometrically at 0.5 s intervals using a video imaging system outfitted with an intensified CCD camera (IonOptix Corp., Milton, MA, USA). A rotating mirror delivered excitation light at 340 or 380 nm. The emitted light was measured at 510 nm, and the average of 29 frames of imaging data was used to calculate numerator and denominator values for determination of 340/380 ratios after background subtraction. [Ca^2+^]_i_ was calculated according to established methods (Grynkiewicz *et al.* 1985). Calibration of raw fura−2 fluorescence values was performed as described (Kang & Holz, 2003) using fura−2 [K^+^]_5_ salt dissolved in calibration buffers from Molecular Probes Inc. (Calcium Calibration Kit 1 with Mg^2+^). Values of *R*_min_ and *R*_max_ were 0.20 and 7.70. Components of the imaging system are illustrated (Supplementary Fig. 1).

**Figure 1:**
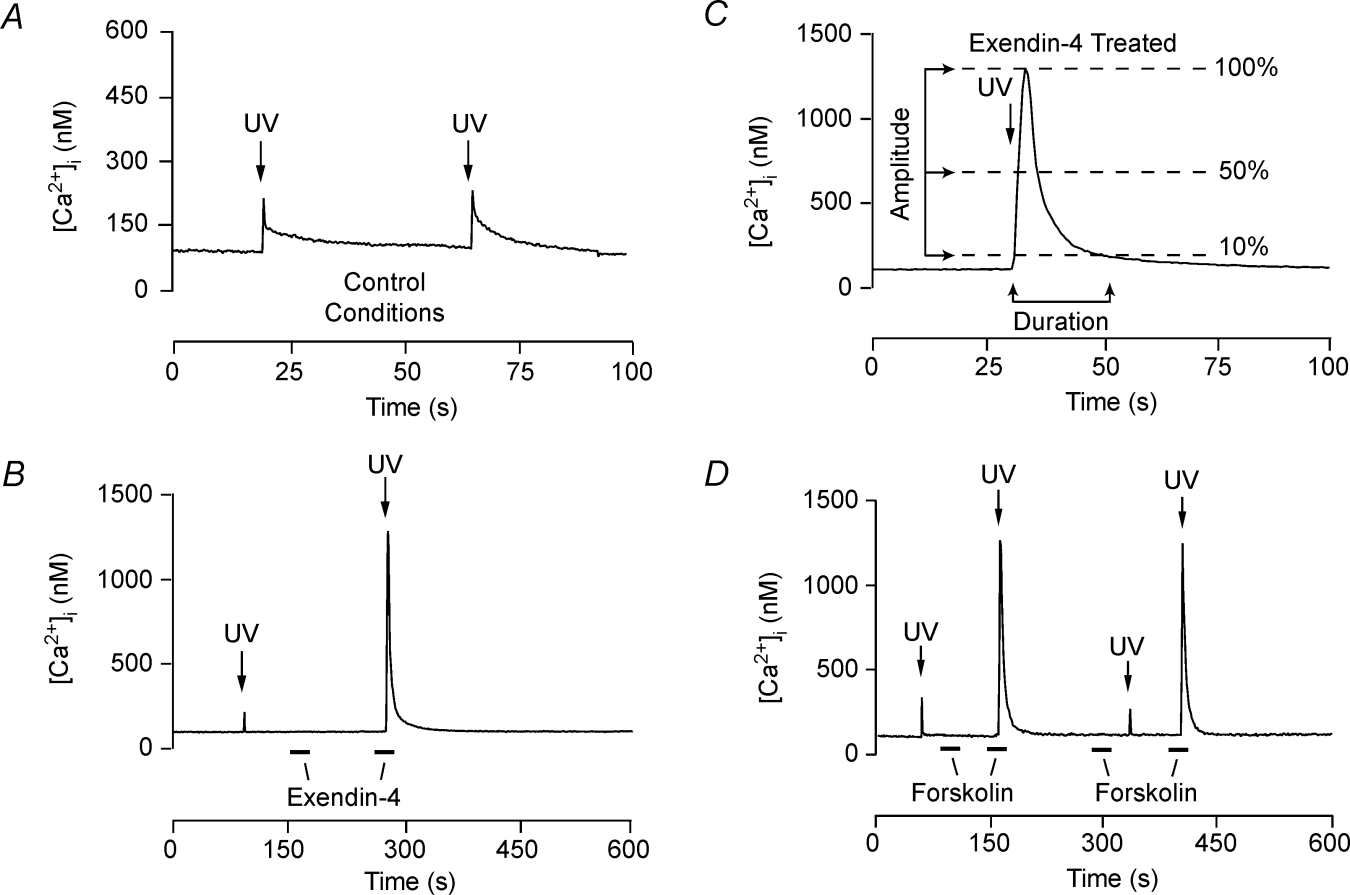
Sensitization of CICR by Ex-4 and forskolin*A,* an INS-1 cell loaded with NP-EGTA was bathed in SES containing 11.1 mm glucose. The uncaging of Ca^2+^ by UV flash photolysis (UV, arrows) produced a small increase of [Ca^2+^]_i_ but did not trigger CICR. *B,* a large transient increase of [Ca^2+^]_i_ (CICR) was observed when UV flash photolysis was performed in the presence but not the absence of Ex-4 (compare *y-*axis scale bars of *A* and *B*). *C,* the action of Ex-4, as illustrated on an expanded time scale. CICR amplitude was determined by subtracting the baseline Ca^2+^ concentration from the Ca^2+^ concentration measured at the peak of the Ca^2+^ transient. Also illustrated are the 50% and 10% threshold values of [Ca^2+^]_i_ that were used when determining the percentage of cells exhibiting CICR (see Methods). *D*, sensitization of CICR by the cAMP-elevating agent forskolin (2 μm). *A* and *D* are from two different cells. *B* and *C* are from the same cell.

### UV flash photolysis for liberation of caged Ca^2+^ and caged IP_3_

Cells were bathed for 60 min at 23°C in SES containing cell-permeable caged Ca^2+^ (NP-EGTA-AM, 5 μm, Molecular Probes, Inc.) or caged IP_3_ (ci-IP_3_/PM, 10 μm). The loading solution also contained 1 μm fura-2-AM, 2% FBS, and 0.02% Pluronic F-127. ci-IP_3_/PM is a photolabile caged IP_3_ related in structure to cm-IP_3_/PM (Li *et al.* 1998). It was synthesized in nine steps from *myo*-inositol in the laboratory of W-H Li, using a procedure similar to that used for making cm-IP_3_/PM (W-H Li, manuscript in preparation). In ci-IP_3_/PM, 2- and 3-hydroxyl groups of *myo*-inositol are protected by an isopropylidene group; whereas in cm-IP_3_/PM, 2- and 3-hydroxyl groups are protected by a methoxymethylene group. Like cm-IP_3_/PM, ci-IP_3_/PM is cell permeable, and once inside cells, induces Ca^2+^ release from intracellular stores upon UV flash photolysis (W-H Li, unpublished results; Wagner *et al.* 2004). Uncaging of caged compounds was achieved using a flash photolysis system (Model JML-C2, Rapp OptoElectronic, Hamburg, Germany). The excitation light of 80 J intensity and 600 μs duration was filtered using a short-pass filter (cut-off 390 nm), and was delivered to the specimen by way of the microscope's objective (Supplementary Fig. 1). The intensity and duration of the flash were minimized so that no measurable photo-bleaching of fura−2 was observed. The intensity of 340 and 380 nm excitation light for detection of fura−2 was also reduced so as to be so low as to produce no measurable uncaging of caged compounds.

### Measurement of endoplasmic reticulum [Ca^2+^] using YC3.3-er

INS-1 cells were transiently transfected with a plasmid encoding yellow cameleon 3.3-er (YC3.3-er) (Griesbeck *et al.* 2001) using Lipofectamine 2000 (Invitrogen-Life Tech.). Cells on glass coverslips were placed within a microperfusion chamber mounted on an inverted microscope (TE-2000, Nikon) and were visualized with a 40× objective (Hara *et al.* 2004). The excitation light (440 nm) was attenuated 50–90% using neutral density filters. Changes in fluorescence emission intensities at 535 nm (citrine; FRET acceptor) and 485 nm (enhanced cyan fluorescent protein, ECFP; FRET donor) were monitored using emission filters mounted on a computer-controlled filter wheel (Lambda 10–2 Optical Filter Changer, Sutter Instruments, Novato, CA, USA). Images (50–100 ms exposure time) were captured with a 16-bit Cascade 650 digital camera (Photometrics, Tucson, AZ, USA) at 2–10 s intervals, and were analysed using MetaMorph/MetaFluor software (Universal Imaging Corp., Downington, PA, USA). Data were expressed as the background subtracted ratios of the FRET acceptor and FRET donor emission intensities monitored at 535 and 485 nm, respectively. Cells were superfused at 32°C in saline of high pH-buffering capacity consisting of (mm): 138 NaCl, 5.6 KCl, 2.6 CaCl_2_, 1.2 MgCl_2_, 25 NaHCO_3,_ 10 Hepes-NaOH (pH 7.40), and 11.1 glucose (Varadi & Rutter, 2004).

### Confocal microscopy for measurement of [Ca^2+^]_i_

Cells plated on 0.15 mm glass coverslips were incubated for 20 min at room temperature in SES containing 5 μm fluo-4 AM (Molecular Probes). The [Ca^2+^]_i_ was imaged using a laser scanning confocal microscope (Leica DM IRE2; Leica Microsystems Heidelberg GmbH) equipped with a 63× water immersion objective (NA 1.2). Fluo-4 was excited at 488 nm using an argon laser. The emitted light passed through a 500 nm dichroic filter for detection using a fluo-4 emission filter (Leica TCS SP2; Leica Microsystems Heidelberg GmbH). Images of the *x*–y optical sections were recorded with a resolution of 512 pixels line^−1^ at 400 Hz. Raster point size was 0.1 μm, with an overall lateral resolution of 0.16 μm. For each data set, 20 *x*–*y* scans of a small cluster of cells were acquired. After the third section was scanned, a test solution was applied via a micropipette. Image analysis was performed using Leica Confocal Software (Version 2.5; Leica Microsystems).

### Microinjection of heparin

Low molecular weight heparin (MW 5000 kDa; Calbiochem) was dissolved in buffer containing (mm): 110 KCl, 10 NaCl, 2 MgCl_2_, 20 Hepes, 5 KH_2_PO (297 mOsmol, pH 7.2). The heparin was injected from Femtotip II needles into individual β cells using a Transjector 5246 microinjection system (Eppendorf, Hamburg, Germany) mounted on a Nikon TE200 inverted microscope. The injection pressure was 100 hPa, the compensation pressure was 33 hPa, and the duration of injection was 0.2 s. In order to identify β cells injected with heparin, the injection solution also contained fluorescein (1 mg ml^−1^; Sigma-Aldrich). The injected cells were then loaded with fura 2 AM and caged compounds. Prior to imaging of Ca^2+^, cells injected with heparin and fluorescein were visualized using an EYFP filter set.

### Epac constructs and transfection protocol

Human wild-type *Epac1* (GenBank Accession No. AAF103905) (de Rooij *et al.* 1998) and dominant negative *Epac1* (R279E) in pcDNA3.1 were obtained from Dr X. Cheng (Galveston, TX) (Qiao *et al.* 2002). Mouse wild-type *Epac2* (cAMPGEFII; Accession No. AB021132) and dominant negative *Epac2* (G114E, G422D) in pSRα were provided by Dr S. Seino (Kobe, Japan) (Ozaki *et al.* 2000; Kashima *et al.* 2001). *Epac1* and *Epac2* cDNAs were subcloned into pCMV2-FLAG (Sigma-Aldrich, USA) to insert the FLAG epitope at the exchange factor's N-terminus. Epac constructs were introduced into INS-1 cells using LipofectAMINE Plus (Invitrogen-Life Tech.). Cells transfected with wild-type or dominant negative *Epac* were identified by cotransfection with pEYFP-N1 (Clonetech, Palo Alto, CA, USA). A 1: 4 molar ratio of pEYFP-N1 relative to Epac plasmid was used in all transfections (Kang *et al.* 2001). EYFP fluorescence was monitored in fura-2-loaded cells 2 days post-transfection, using 513 nm excitation and 527 nm emission filters. Once an EYFP-positive cell was identified as having been transfected, the filter set was manually switched to a fura-2 filter set, allowing ratiometric determinations of [Ca^2+^]_i_. Control experiments demonstrated that less than 1% crossover existed between fura-2 and EYFP when using filter sets selective for each reporter (see Supplementary Figs 2–4).

### Detection of recombinant Epac immunoreactivity

Whole cell lysates of transfected INS-1 cells expressing recombinant Epac were dissolved in 1× Laemmli's sample buffer, boiled for 5 min, centrifuged to remove unsolubilized material, and resolved by SDS-PAGE using 4% stacking and 12% resolving gels. The resolved Epac proteins were transferred to Immobilon-P PVDF membrane (Millipore, Bedford, MA, USA) by electrophoretic transfer (120 V, 1 h). Western immunoblot analyses were performed using mouse anti-FLAG monoclonal primary antiserum (Sigma-Aldrich; 1: 1000 dilution) in combination with goat antimouse polyclonal secondary antiserum (1: 5000 dilution) conjugated to horseradish peroxidase (Sigma-Aldrich).

### CRE-Luc reporter assay

Luciferase activity was measured in lysates of INS-1 cells transfected with CRE-Luc (Stratagene, La Jolla, CA, USA) as described (Chepurny *et al.* 2002,Chepurny & Holz, 2002). This construct allows expression of luciferase to be regulated by cAMP response elements (CREs) located within a minimal promoter. Monolayers of INS–1 cells were exposed to test substances for 4 h, lysed and assayed for luciferase-catalysed photoemissions using a luciferase assay kit (Promega, Madison, WI, USA) and a luminometer allowing automated application of solutions containing ATP and luciferin (Model TR-717, Perkin Elmer Applied Biosystems, Foster City, CA, USA). Experiments were carried out in triplicate. Statistical analyses were performed using an ANOVA test combined with Fisher's PLSD test.

### Sources of reagents and application of test substances

Exendin-4, forskolin, caffeine, ryanodine, and thapsigargin were from Sigma-Aldrich. H-89 was from Calbiochem (San Diego, CA, USA). 8-pCPT-cAMP, 6-Bnz-cAMP, and 8-pCPT-2′-*O*-Me-cAMP were from BioLog Life Science (Bremen, Germany). Test solutions dissolved in SES were added to the bath solution or were applied to individual cells from glass ‘puffer’ micropipettes (type 1B150-6; World Precision Institute Inc., Sarasota, FL, USA) using a pressure ejection system (PicoSpritzer II, General Valve Corp., NJ, USA) as described (Holz *et al.* 1993).

### Statistical analyses of CICR

Population studies were performed at the single-cell level in order to determine the percentage of cells exhibiting CICR under conditions in which cells were treated with pharmacological agents added directly to the bath and ‘puffer’ pipette solutions. A coverslip with adherent cells served as a control, while a ‘sister’ culture served as the test. At least 10 cells (coverslip)^−1^ were selected one at a time in random order to determine basal [Ca^2+^]_i_ and CICR amplitude. CICR in INS-1 cells was defined as a transient increase of [Ca^2+^]_i_, the duration of which did not exceed 30 s when measured at the 10% amplitude cut-off (Fig. 1*C*). An additional requirement was that the increase of [Ca^2+^]_i_ for INS-1 cells must have exceeded 200 nm when measured at the 50% amplitude cut-off (Fig. 1*C*). Because CICR in mouse β cells tended to be of smaller amplitude, these criteria were modified so that the increase of [Ca^2+^]_i_ must have exceeded 125 nm when measured at the 50% amplitude cut-off. Each experiment was performed in triplicate, and statistical analyses were performed using the ANOVA test combined with Fisher's PLSD test.

## Results

### Sensitization of CICR by exendin-4 and forskolin

To assess the properties of CICR in INS-1 cells, we performed UV flash photolysis to uncage Ca^2+^ from NP-EGTA, a photolabile Ca^2+^ chelator. Prior to the loading of cells with NP-EGTA, the resting [Ca^2+^]_i_ was 135 ± 65 nm (mean ±s.d.; *n*= 20 cells). After loading with NP-EGTA, there was a statistically significant reduction of [Ca^2+^]_i_ to 91 ± 5 nm (*P* < 0.001; *n*= 20 cells). A 600 μsec flash of UV light produced a small increase of [Ca^2+^]_i_ in these cells loaded with NP-EGTA (122 ± 26 nm increase; *n*= 30 cells) (Fig. 1*A*). This increase of [Ca^2+^]_i_ recovered to its original baseline and was fully repeatable (Fig. 1*A*). No such increase of [Ca^2+^]_i_ was measured in cells not loaded with NP-EGTA (*n*= 20 cells; data not shown). It may be concluded that the increase of [Ca^2+^]_i_ depicted in Fig. 1*A* resulted from the uncaging of Ca^2+^.

When the GLP-1-R agonist exendin-4 (Ex-4, 1 nm) was applied to single INS-1 cells loaded with NP-EGTA, no increase of [Ca^2+^]_i_ was measured (Fig. 1*B*). In contrast, a large and transient increase of [Ca^2+^]_i_ (CICR) was observed when Ex-4 was applied simultaneous with the uncaging of Ca^2+^ (Fig. 1*B*). As summarized in Table 1*A*, this sensitizing action of Ex-4 to promote CICR was observed in 17 of 24 cells tested. The mean amplitude of the Ca^2+^ spike measured under these conditions was 1110 ± 176 nm, and the mean duration measured at the 50% amplitude cut-off was 5.1 ± 1.3 s (*n*= 15 cells). Sensitization of CICR was also observed when UV flash photolysis was performed in the presence of cAMP-elevating agent forskolin (2 μm) (Fig. 1*D*). This action of forskolin was repeatable and was observed in 23 of 30 cells tested.

**Table 1:** Ryanodine sensitivity of CICR

Treatment	% of cells (*n*) exhibiting CICR (no ryanodine)	Mean ±s.d. increase of [Ca^2+^]_i_with UV flash (nm)	% of cells (*n*) exhibiting CICR (with ryanodine)
A INS-1 cells			
Ex-4	71 (24)	1119 ± 176	22 (18)^*^
8-pCPT-cAMP	69 (13)	1046 ± 156	24 (17)^*^
Caffeine	74 (27)	1252 ± 216	18 (17)^*^
B Mouse β cells			
Forskolin	73 (11)	422 ± 63	33 (12)^*^
Caffeine	67 (12)	487 ± 79	27 (11)^*^

Values of *n* shown in parentheses. The concentrations of test substances were: Ex-4 (1 nm), 8-pCPT-cAMP (100 μm), caffeine (1 mm), and forskolin (2 μm). Each test substance was applied for 30 s to individual cells while performing the uncaging of Ca^2+^. The mean increase of [Ca^2+^]_i_ refers to cells exhibiting CICR. Ryanodine (10 μm) was added directly to the bath solution and cells were allowed to equilibrate with ryanodine for 10–15 min at 34°C. ^*^The percentage of cells exhibiting CICR in the presence of ryanodine was significantly different from that in the absence of ryanodine (*P* < 0.001).

### Sensitization of CICR by cAMP analogues

**Figure 2:**
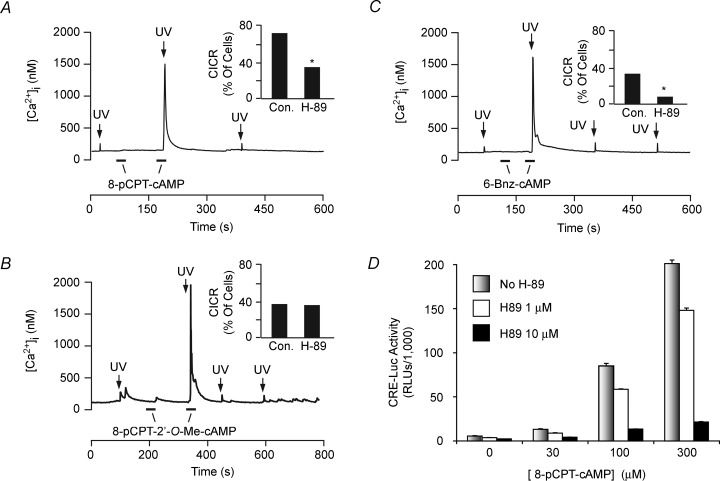
Sensitization of CICR by cAMP analogues The uncaging of Ca^2+^ produced an increase of [Ca^2+^]_i_ in INS-1 cells treated with 8-pCPT-cAMP (*A*), 8-pCPT-2′-*O*-Me-cAMP (*B*), or 6-Bnz-cAMP (*C*). Horizontal bars indicate the time at which 100 μm of each cAMP analogue was administered. Insets of *A*–*C* illustrate the percentage of cells (*n*= 20 cells per cAMP analogue) exhibiting CICR under control conditions or under conditions in which cells were treated with the H-89 (10 μm). *P < 0.01 compared to control. *D,* the activity of CRE-Luc in INS-1 cells was stimulated by 8-pCPT-cAMP and the action of 8-pCPT-cAMP was inhibited by H-89. Luciferase activity is expressed as relative light units (RLUs).

cAMP and Ca^2+^ may interact to generate CICR in INS-1 cells. To evaluate this possibility, we examined whether cell-permeant cAMP analogues sensitize the CICR mechanism of INS-1 cells in a manner analogous to that described for Ex-4 and forskolin. When NP-EGTA-loaded INS-1 cells were exposed to 8-pCPT-cAMP (100 μm), an activator of both PKA and Epac, the uncaging of Ca^2+^-triggered CICR (Fig. 2*A*, Table 1*A*). This action of 8-pCPT-cAMP was reduced but not blocked by treatment with H-89 (10 μm), a PKA inhibitor (Fig. 2*A*; inset). CICR was also observed when cells were exposed to 8-pCPT-2′-*O*-Me-cAMP (100 μm), a cAMP analogue active at Epac only (Fig. 2*B*), or 6-Bnz-cAMP (100 μm), an analogue active at PKA only (Fig. 2*C*). As predicted, 8-pCPT-2′-*O*-Me-cAMP remained effective in cells treated with H-89, whereas the action of 6-Bnz-cAMP was nearly abrogated (cf. Fig. 2*B* and *C*; insets). Given that INS-1 cells express mRNA corresponding to Epac1 and Epac2 (Leech *et al.* 2000), such findings are expected if the sensitizing action of cAMP is mediated not only by PKA but also by Epac. To confirm the efficacy of H-89 as an inhibitor of PKA, the activity of a PKA-regulated luciferase reporter (CRE-Luc) was assessed in transfected INS-1 cells. Under conditions of 8-pCPT-cAMP treatment, both the basal and stimulated activities of CRE-Luc were inhibited by H-89 (Fig. 2*D*).

### Exendin-4 exerts its action via PKA and Epac

**Figure 3:**
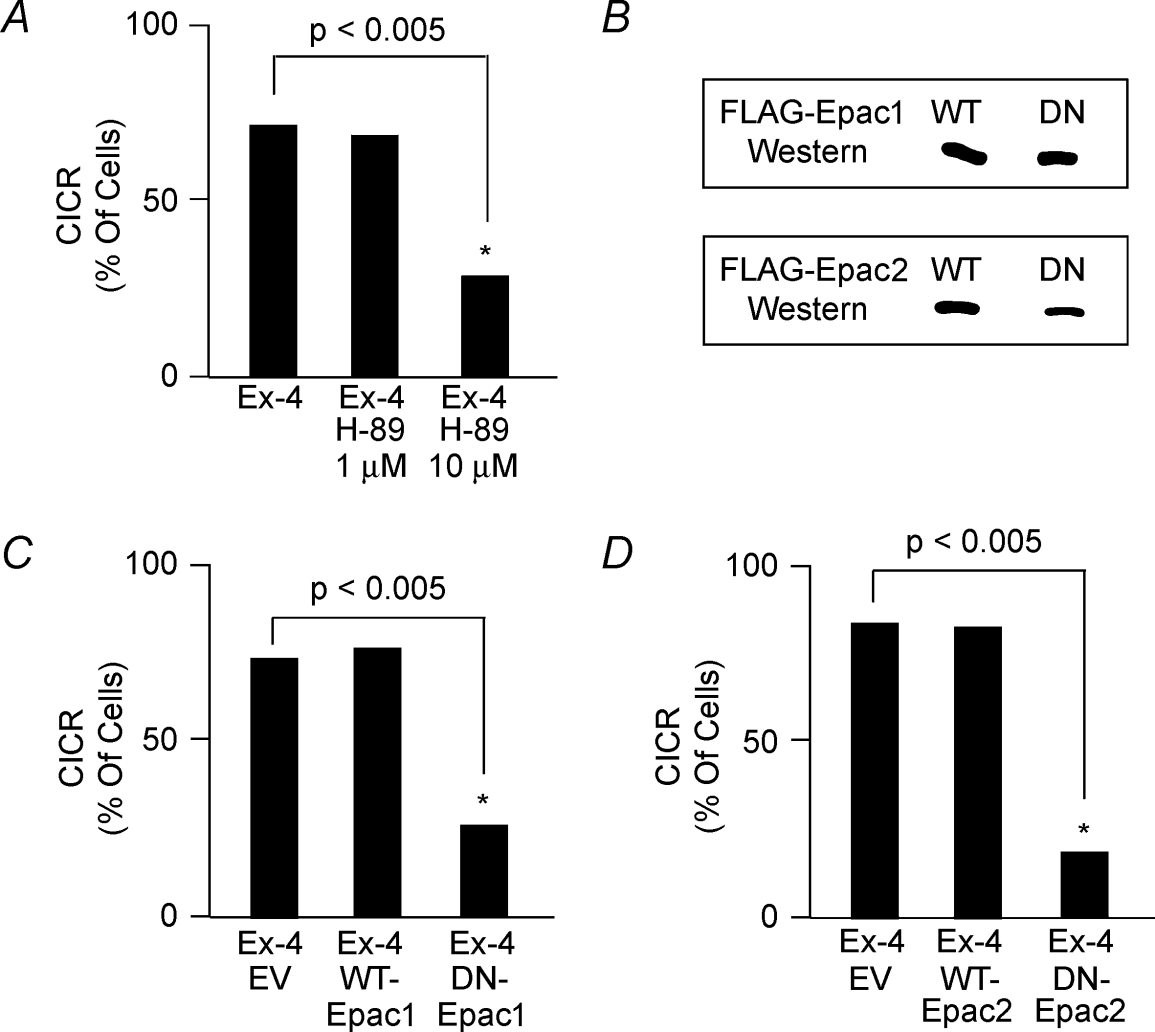
The action of Ex-4 is mediated by PKA and Epac *A,* the percentage of INS-1 cells exhibiting CICR was determined under conditions of UV flash photolysis in which cells were treated with Ex-4 (1 nm) in the absence or presence of H-89. *B*, western blot analyses confirmed that wild-type (WT) and dominant negative (DN) FLAG-Epac1 and FLAG-Epac2 were expressed in transiently transfected INS-1 cells. *C* and *D,* transient transfection of INS-1 cells with DN FLAG-Epac1 or FLAG-Epac2 reduced the percentage of cells exhibiting CICR under conditions of UV flash photolysis in which cells were treated with Ex-4 (1 nm). No such inhibitory effect was observed after transfection of cells with empty vector (EV) or wild-type (WT) FLAG-Epac constructs. For each histogram bar a minimum of 20 cells was tested. *P < 0.005).

We next sought to ascertain if it is PKA or Epac that mediates the cAMP-dependent mobilization of Ca^2+^ by Ex-4. To this end, the action of Ex-4 was evaluated after treatment of INS-1 cells with H-89 or after transfection with dominant negative isoforms of Epac that do not bind cAMP (Holz, 2004*a*). Population studies demonstrated that H-89 exerted partial inhibitory effects when evaluating its ability to suppress CICR. Whereas a low concentration of H-89 (1 μm) was without effect, a higher concentration (10 μm) reduced the percentage of cells responding to Ex-4 by 60% (Fig. 3*A*). The ineffectiveness of 1 μm H-89 in this assay of CICR is notable, because this concentration of H-89 reduced CRE-Luc activity by 33% (Fig. 2*D*). Therefore, there may exist a PKA-independent signalling pathway by which Ex-4 exerts its sensitizing action. This is likely to be the case because the action of Ex-4 was inhibited by overexpression (see immunoblot, Fig. 3*B*) of dominant negative (DN) FLAG epitope-tagged Epac1 (Fig. 3*C*) or Epac2 (Fig. 3*D*). These DN Epacs incorporate inactivating amino acid substitutions within their cAMP-binding domains (Holz, 2004*a*). Importantly, overexpression of wild type (WT) FLAG-Epac1 or FLAG-Epac2 failed to confer such an inhibitory effect (Figs 3*C* and *D*).

### Ryanodine and thapsigargin-sensitive Ca^2+^ stores contribute to CICR

**Figure 4:**
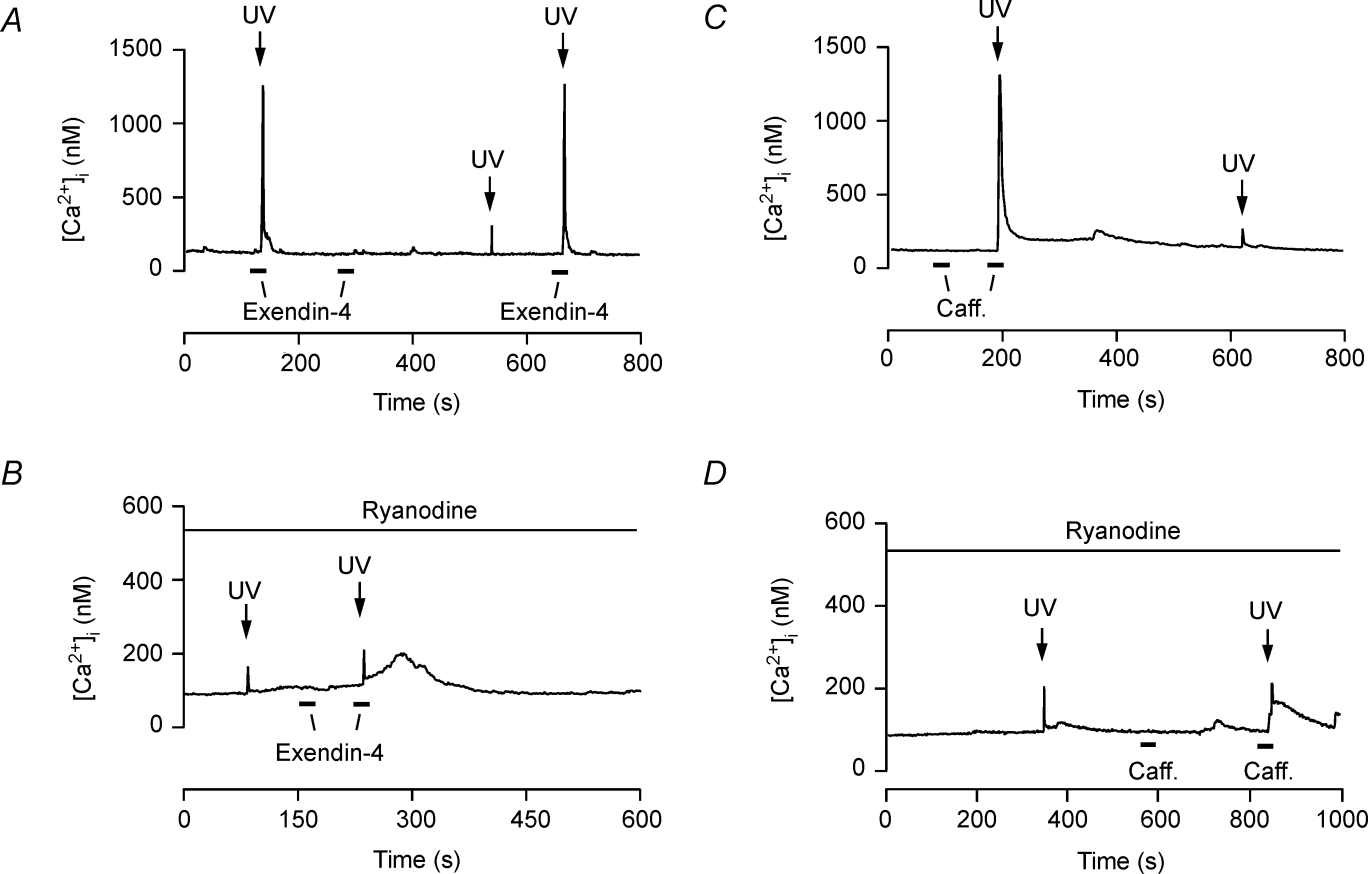
Exendin-4 and caffeine mobilize Ca^2+^ from ryanodine-sensitive Ca^2+^ stores. *A* and *B,* the uncaging of Ca^2+^-stimulated CICR under conditions in which INS-1 cells were treated with Ex-4 (1 nm) in the absence (*A*) but not the presence (*B*) of ryanodine. *C* and *D,* sensitization of CICR by caffeine (1 mm, Caff.) was blocked by treatment of INS-1 cells with ryanodine. In *B* and *D*, ryanodine (10 μm) was added to the bath solution for 10–20 min prior to the uncaging of Ca^2+^. Each panel illustrates representative findings obtained for a minimum of 10 cells studied.

**Figure 5:**
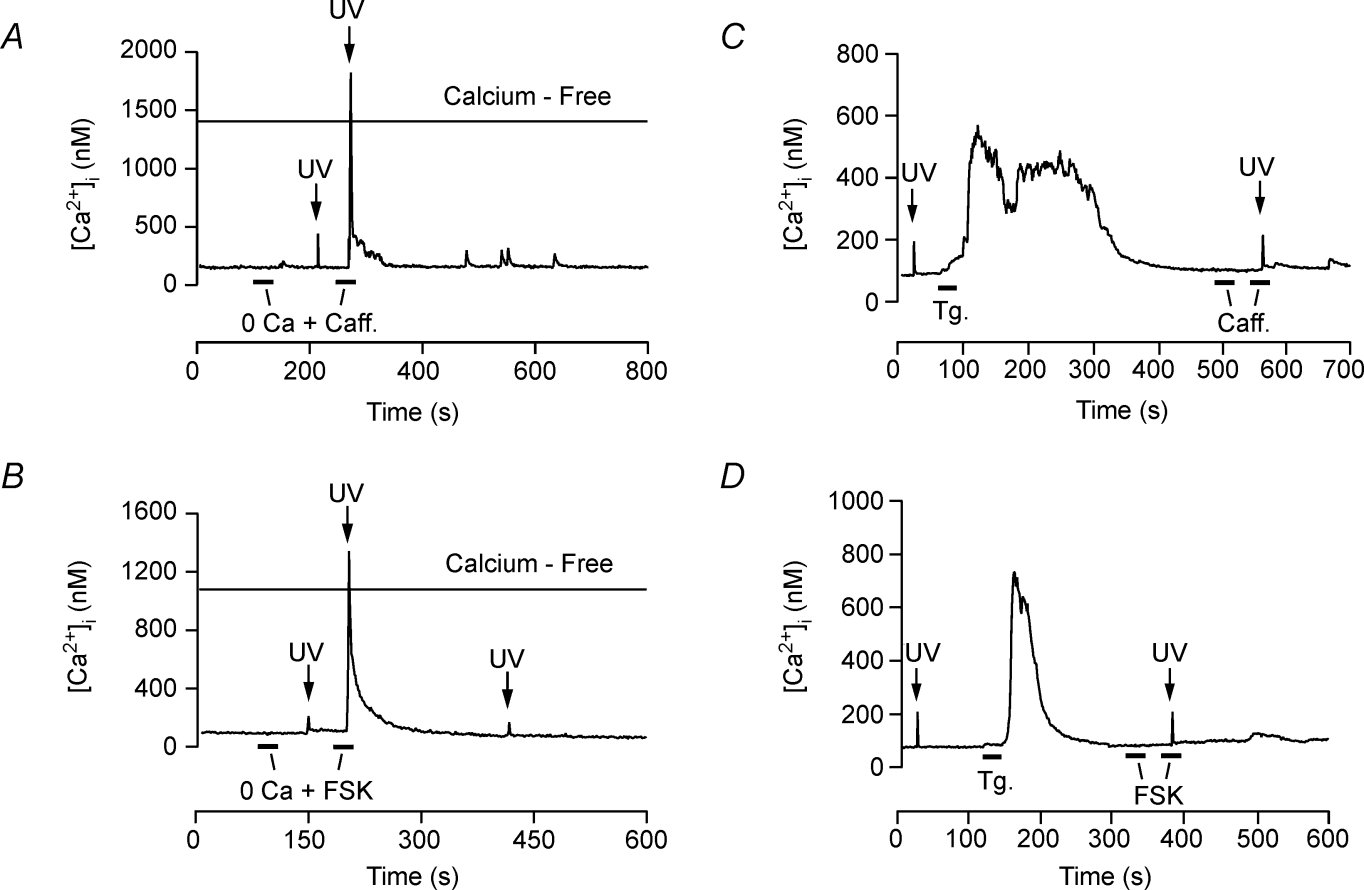
Extracellular Ca^2+^ independence and thapsigargin sensitivity of CICR. *A* and *B,* treatment of INS-1 cells with caffeine (1 mm, Caff.) or forskolin (2 μm; FSK) allowed for the appearance of CICR under conditions in which cells were bathed in a nominally Ca^2+^-free solution (0 Ca). *C* and *D,* treatment of INS-1 cells with thapsigargin (1 μm, Tg.) stimulated an increase of [Ca^2+^]_i_ and abolished CICR measured under conditions in which cells were exposed to caffeine or forskolin. Each panel illustrates representative findings obtained for a minimum of 10 cells studied.

A 10–15 min pretreatment of INS-1 cells with ryanodine (10 μm) reduced the percentage of INS-1 cells exhibiting CICR under conditions of UV flash photolysis. This was the case when the uncaging of Ca^2+^ was performed in the presence of Ex-4 (1 nm) or 8-pCPT-cAMP (100 μm) (Fig. 4*A* and *B*; Table 1*A*). Caffeine *(*1 mm) mimicked the action of Ex-4 (Fig. 4*C*), and the action of caffeine was also inhibited by ryanodine (Fig. 4*D*; Table 1*A*). To validate that an intracellular source of Ca^2+^ was mobilized as a consequence of CICR, the actions of caffeine and forskolin were examined under conditions in which the SES was nominally Ca^2+^ free. Under such conditions, caffeine (1 mm) and forskolin (2 μm) allowed for the appearance of CICR (Fig. 5*A* and *B*). The source of Ca^2+^ mobilized by caffeine and forskolin included thapsigargin-sensitive Ca^2+^ stores, because the actions of both agents were abrogated by pretreatment with thapsigargin (1 μm) (Fig. 5*C* and *D*).

### Caffeine mobilizes endoplasmic reticulum Ca^2+^

**Figure 6:**
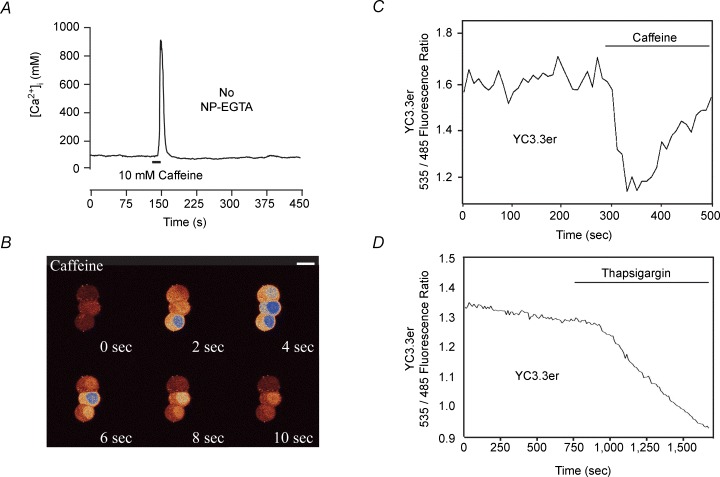
Mobilization of endoplasmic reticulum Ca^2+^. *A,* a high concentration of caffeine (10 mm) stimulated an increase of [Ca^2+^]_i_ in an INS-1 cell not loaded with NP-EGTA. *B,* this action of caffeine appeared as an increase of [Ca^2+^]_i_ in the cytosol and nucleus. The photomontage illustrates the time course of the response to caffeine (10 mm) applied at time = 0 s. The gain on the imaging system's detector was increased to allow measurement of cytosolic fluorescence (orange) under conditions in which an apparently large increase of nuclear Ca^2+^ concentration saturated the detector (blue). Scale bar, 10 μm. *C,* FRET-based detection of YC3.3er in INS-1 cells demonstrated that caffeine (10 mm) reduced ER Ca^2+^ concentration. *D,* a decrease of ER Ca^2+^ concentration was also observed during treatment of an INS-1 cell with thapsigargin (5 μm).

A high concentration of caffeine (10 mm) produced a transient increase of [Ca^2+^]_i_ in INS-1 cells not loaded with NP-EGTA. This action of caffeine was fast in onset and recovered to baseline within 10–20 s (Fig. 6*A*). The increase of [Ca^2+^]_i_ was measured in the cytoplasm and also the nucleus (Fig. 6*B*). The source of Ca^2+^ mobilized included the endoplasmic reticulum (ER), as demonstrated using INS-1 cells expressing a cameleon Ca^2+^ reporter (YC3.3-er) targeted to the ER. When 10 mm caffeine was administered to these cells, a transient decrease of 535/485 nm emission ratio was detected (Fig. 6*C*). This signifies a decrease of ER calcium concentration ([Ca^2+^]_ER_) (Griesbeck *et al.* 2001). The [Ca^2+^]_ER_ of INS-1 cells was also lowered when these cells were exposed to thapsigargin (Fig. 6*D*).

### Sensitization of CICR by cAMP-elevating agents in mouse β cells

**Figure 7:**
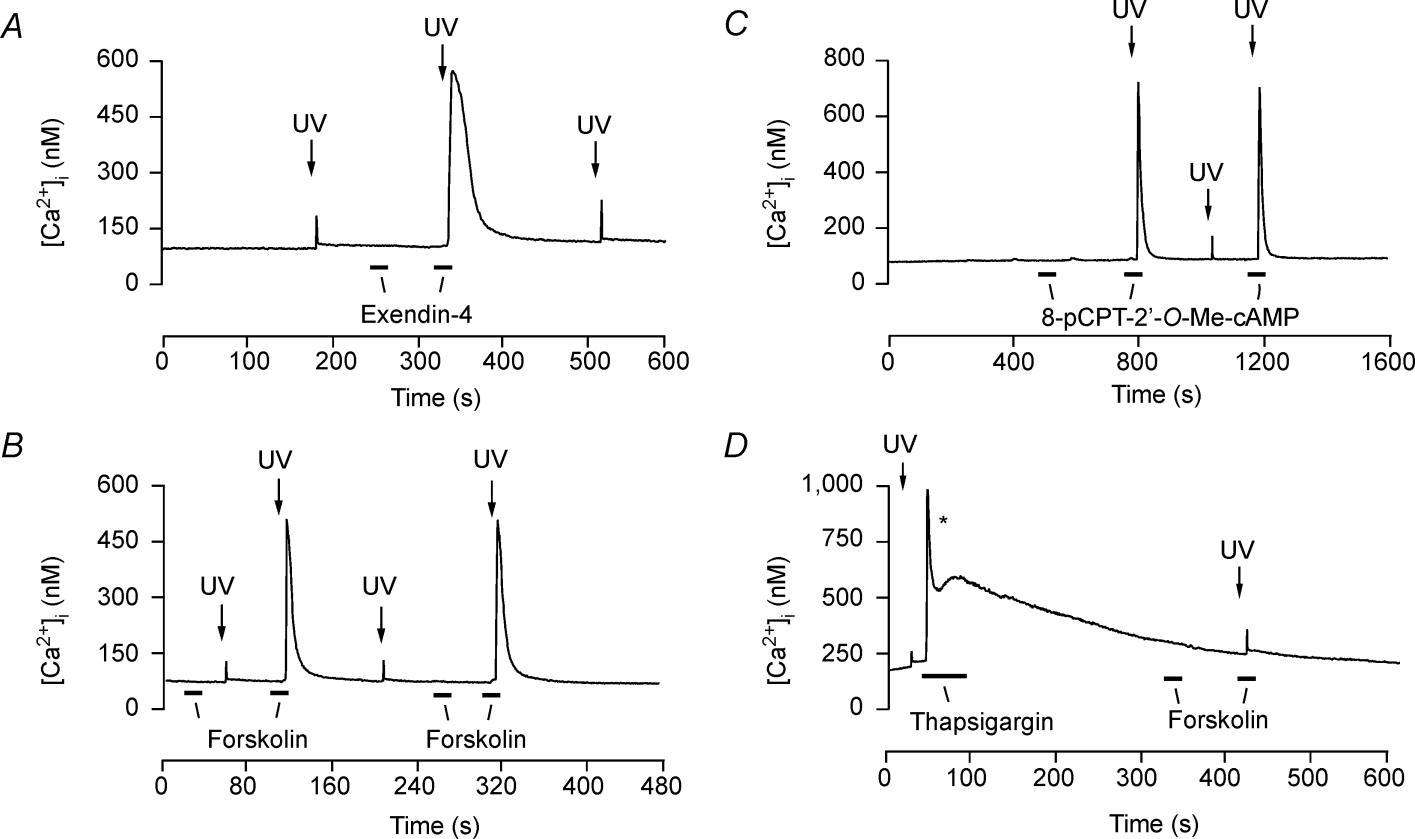
cAMP-elevating agents sensitize CICR in mouse β cells. *A,* uncaging of Ca^2+^ in a mouse β cell produced a small increase of [Ca^2+^]_i_ in the absence of Ex-4, whereas CICR was observed in the presence of Ex-4 (1 nm). *B* and *C,* CICR was also observed when Ca^2+^ was uncaged in the presence of forskolin (2 μm) or 8-pCPT-2′-*O*-Me-cAMP (100 μm). *D,* the action of forskolin was abolished when a mouse β cell was pretreated with thapsigargin (1 μm). Note that thapsigargin, alone, stimulated a fast transient increase of [Ca^2+^]_i_ (*). Each panel illustrates representative findings obtained for a minimum of 10 cells studied.

To examine how cAMP-elevating agents influence CICR in mouse β cells, UV flash photolysis was performed under conditions of NP-EGTA loading identical to that described for INS-1 cells. Prior to loading, the resting [Ca^2+^]_i_ was 104 ± 29 nm (*n*= 25 cells). This value decreased to 87 ± 14 nm (*n*= 22 cells) in NP-EGTA-loaded cells (*P* < 0.001; *n*= 20 cells). The uncaging of Ca^2+^ in β cells generated a small increase of [Ca^2+^]_i_ but did not initiate CICR (Fig. 7*A*; 55 ± 11 nm increase; *n*= 17 cells). In these same cells, application of Ex-4 (1 nm), forskolin (2 μm), or the Epac-selective cAMP analogue 8-pCPT-2′-*O*-Me-cAMP (100 μm) failed to alter resting [Ca^2+^]_i_ (Fig. 7*A*–*C*). However, all three cAMP-elevating agents allowed for the appearance of CICR under conditions of UV flash photolysis (Fig. 7*A*–*C*; Table 1*B*). 8-pCPT-2′-*O*-Me-cAMP (100 μm) was highly effective in this assay. It allowed for the appearance of CICR in 8 of 15 cells tested. Similar to findings obtained with INS-1 cells, no sensitization of CICR by forskolin was observed when mouse β cells were treated with thapsigargin (Fig. 7*D*).

### Characterization of Ca^2+^ release channels targeted by cAMP in mouse β cells

To assess whether CICR sensitized by forskolin resulted from activation of RYR, the action of ryanodine was examined. A role for RYR is indicated because pretreatment with ryanodine (10 μm) rendered forskolin less effective in the assay of β cell CICR reported here (Table 1*B*). Furthermore, caffeine (1 mm), a sensitizer of RYR, allowed for the appearance of CICR under conditions of UV flash photolysis. This action of caffeine was also diminished by ryanodine (Table 1*B*).

**Figure 8:**
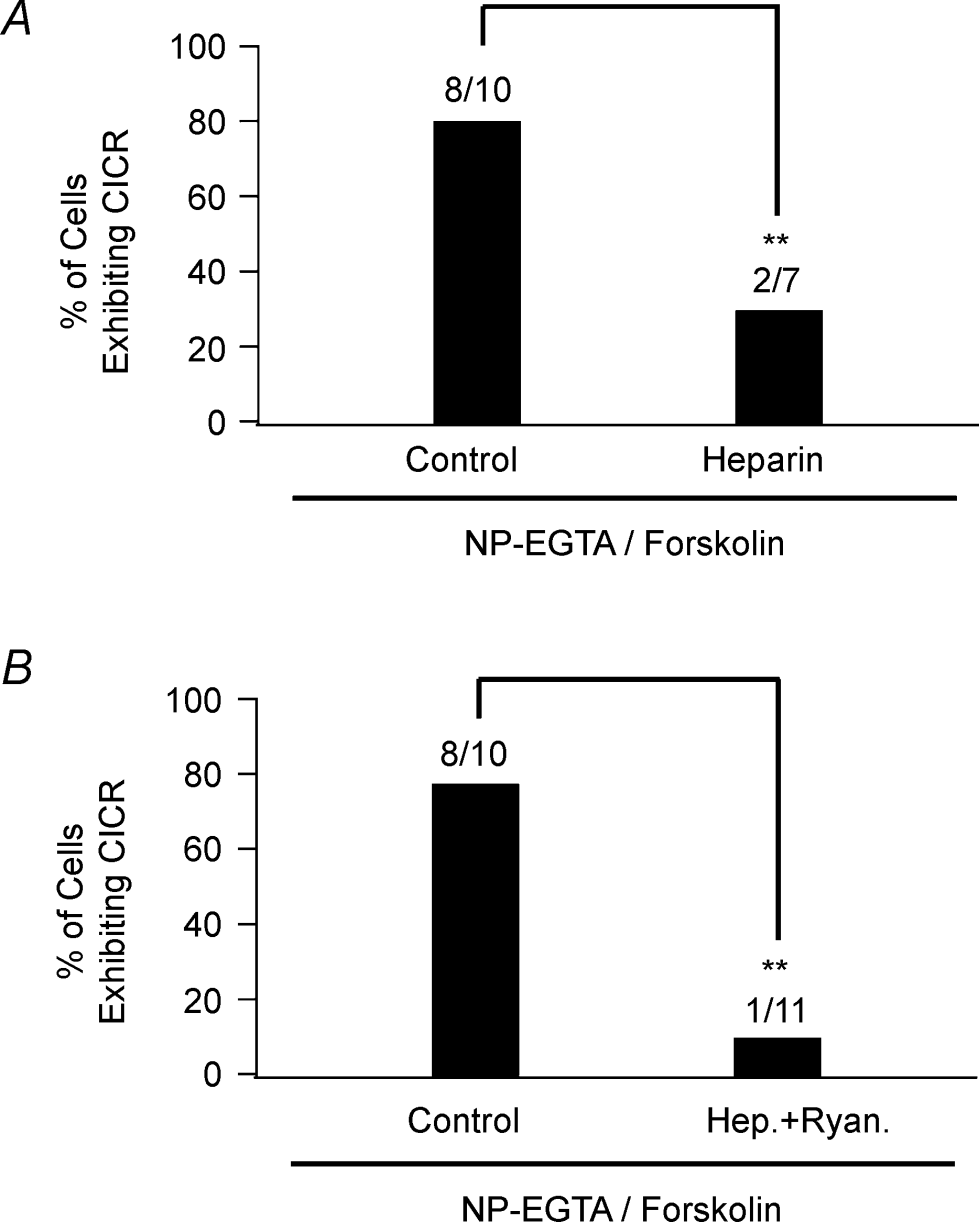
Antagonism of CICR by heparin and ryanodine in mouse β cells. *A,* antagonism of CICR by intracellularly administered heparin in mouse β cells loaded with NP-EGTA. The uncaging of Ca^2+^ by UV flash photolysis was performed under conditions in which β cells were exposed to forskolin (2 μm). Control conditions refer to cells injected with a solution containing fluorescein but not heparin (see Methods). *B,* the sensitization of CICR by forskolin was nearly abolished under conditions in which heparin (Hep.)-injected β cells were also treated with ryanodine (10 μm; Ryan.). Numbers above each histogram bar indicate the fraction of cells exhibiting CICR. **P < 0.01.

Because ryanodine-resistant CICR was sometimes observed (Table 1*B*), a sensitizing action of cAMP at the IP_3_-R may also exist in β cells. Therefore, we assessed the efficacy of an IP_3_-R inhibitor (low molecular weight heparin, 100 mg ml^−1^; Ehrlich *et al.* 1994) administered intracellularly under conditions of NP-EGTA loading and UV flash photolysis. Administration of heparin led to a reduction in the percentage of forskolin-treated β cells exhibiting CICR (Fig. 8*A*). Moreover, CICR was nearly abolished after combined treatment with heparin and ryanodine, thereby demonstrating that these two inhibitors of Ca^2+^ release channels acted in an additive manner (Fig. 8*B*).

**Figure 9:**
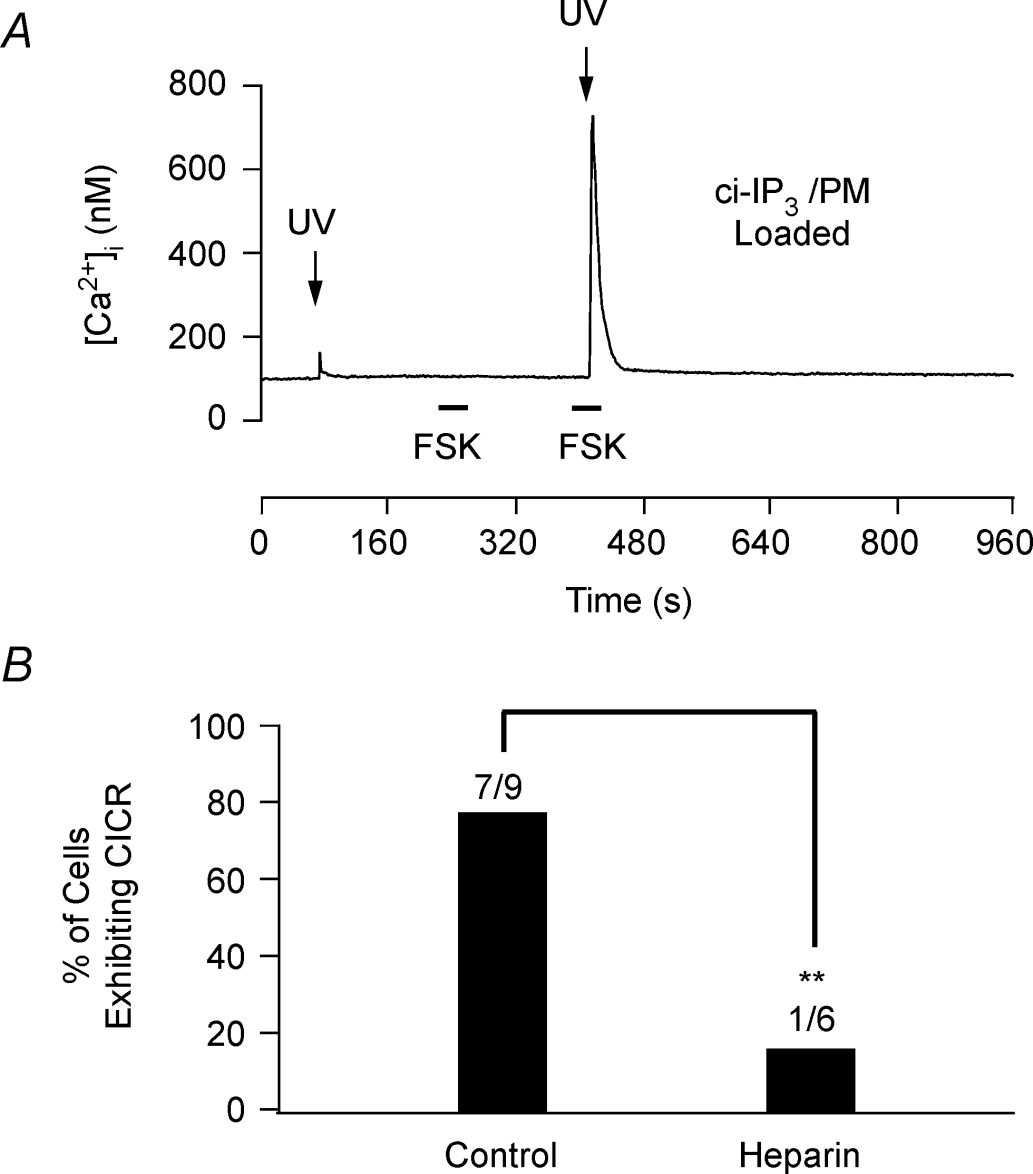
IP_3_-R-mediated CICR sensitized by forskolin in mouse β cells. *A,* low-intensity UV flash photolysis produced a small increase of [Ca^2+^]_i_ (61 ± 17 nm; *n*= 15) in a mouse β cell loaded with membrane-permeant caged IP_3_ (ci-IP_3_/PM). When this cell was treated with forskolin (2 μm; FSK), the uncaging of IP_3_ generated a large Ca^2+^ spike (528 ± 96 nm; *n*= 14). *B*, sensitization of IP_3_-R-mediated CICR by forskolin was inhibited by intracellularly administered heparin. The uncaging of IP_3_ in forskolin-treated β cells generated CICR in 78% of cell tested, whereas this percentage was reduced to 17% under conditions of intracellular heparin administration. Numbers above each histogram bar indicate the fraction of cells exhibiting CICR. **P < 0.01.

To confirm the specificity with which heparin exerted its inhibitory effect, β cells were loaded with a cell-permeant caged IP_3_ (ci-IP_3_/PM). The uncaging of IP_3_ generated a small increase of [Ca^2+^]_i_ under conditions of low-intensity UV flash photolysis, but it did not trigger CICR (Fig. 9*A*). When β cells were exposed to forskolin (2 μm), the small increase of [Ca^2+^]_i_ was converted into a large Ca^2+^ spike (Fig. 9*A*). This CICR sensitized by forskolin required the activity of IP_3_ receptors, because it was abolished after administration of heparin (Fig. 9*B*). It may be concluded that in β cells, IP_3_-R-mediated CICR complements RYR-mediated CICR, and that both processes are cAMP regulated.

## Discussion

### Physiological significance of CICR in β cells

Prior studies demonstrate that cAMP-elevating agents mobilize Ca^2+^ in β cells and insulin-secreting cell lines (Islam *et al.* 1998; Holz *et al.* 1999; Kang *et al.* 2001, 2003; Kang & Holz, 2003; Dyachok & Gylfe, 2004; Dyachok *et al.* 2004). Ca^2+^ mobilized in this manner acts as a direct stimulus for exocytosis of insulin (Kang & Holz, 2003; Kang *et al.* 2003; Dyachok & Gylfe, 2004; reviewed by Holz, 2004*b*). Thus, exocytosis in β cells is not simply dependent on influx of Ca^2+^ through VDCCs, but is also dependent on the interaction of Ca^2+^ and cAMP to promote CICR. In this regard, new studies provide evidence for the existence of a highly Ca^2+^-sensitive pool (HCSP) of secretory granules in β cells (Wan *et al.* 2004; Yang & Gillis, 2004). These granules are released under conditions in which the [Ca^2+^]_i_ increases to low micromolar levels. Since the mechanism of cAMP-dependent Ca^2+^ mobilization reported here generates an increase of [Ca^2+^]_i_ in the 0.5–2 μm range, it may serve as an adequate stimulus for exocytosis of the HCSP.

### Second messenger coincidence detection in β cells

Here we describe an experimental strategy by which the existence of cAMP-regulated CICR in β cells may be validated. The strategy relies on the use of caged Ca^2+^ and UV flash photolysis to produce a small increase of [Ca^2+^]_i_. Under these conditions, the uncaging of Ca^2+^ triggers CICR when Ca^2+^ release channels are sensitized by cAMP-elevating agents. Of particular note is our demonstration that the uncaging of Ca^2+^ fails to generate CICR in the absence of cAMP-elevating agents. This key observation leads us to conclude that a simultaneous increase of intracellular Ca^2+^ and cAMP concentrations is a necessary prerequisite for the initiation of CICR in β cells. Fundamentally, this is a process of second messenger coincidence detection, and it results from dual stimulatory actions of Ca^2+^ and cAMP at Ca^2+^ release channels. These Ca^2+^ release channels correspond to RYR and the IP_3_-R, as demonstrated by antagonism of CICR following application of ryanodine or heparin.

IP_3_-R-mediated Ca^2+^ mobilization is also demonstrated through our use of ci-IP_3_/PM, a membrane-permeant caged IP_3_ that activates the IP_3_-R in a selective manner. Under conditions in which β cells are treated with forskolin, the uncaging of IP_3_ stimulates CICR. This action of forskolin might reflect cAMP-dependent sensitization of the IP_3_-R. Alternatively, cAMP may sensitize RYR to stimulatory effects of Ca^2+^ released from activated IP_3_ receptors. If so, heparin would act at the IP_3_-R to prevent the small rise of [Ca^2+^]_i_ that acts as an initiator of CICR mediated by RYR. Indeed, the mobilization of Ca^2+^ by IP_3_ in pancreatic acinar cells is reported to trigger CICR mediated by ryanodine receptors (Straub *et al.* 2000; Ashby *et al.* 2002). Thus, it will be of interest to ascertain whether there also exist functional interactions between IP_3_ receptors and RYR in β cells.

### Coincidence detection may explain context specificity

Prior studies of β cells demonstrate that CICR occurs in a context-specific manner (Lemmens *et al.* 2001; Bruton *et al.* 2003). CICR is only observed when β cells are equilibrated in elevated concentrations of extracellular glucose. Metabolism of glucose by β cells promotes the filling of ER Ca^2+^ stores (Maechler *et al.* 1999; Tengholm *et al.* 1999), and it also produces an increase of [Ca^2+^]_i_ (Henquin, 2000). Therefore, under conditions in which β cells are exposed to glucose, two necessary conditions are met. ER Ca^2+^ stores are full and the cytosolic Ca^2+^ concentration is elevated sufficiently to allow CICR to be generated when Ca^2+^ release channels are sensitized by cAMP. Context specificity of this type is likely to be of physiological significance, because it may explain, at least in part, why the insulin secretagogue action of GLP-1 is strictly dependent on exposure of β cells to glucose (Kieffer & Habener, 1999).

### RYR as a determinant of β cell function

Because insulin-secreting cells express RYR, albeit at low levels (Takasawa *et al.* 1998; Gamberucci *et al.* 1999; Holz *et al.* 1999; Islam, 2002; Lee *et al.* 2002; Mitchell *et al.* 2003; Beauvois *et al.* 2004; Johnson *et al.* 2004*a*,*b*), it is not surprising that caffeine, a sensitizer of RYR, recapitulates the Ca^2+^-mobilizing action of Ex-4 reported here. This action of caffeine is inhibited by ryanodine, thereby confirming the existence of CICR mediated by RYR. Such findings are consistent with prior studies demonstrating ryanodine and caffeine-sensitive mobilization of Ca^2+^ in β cells or β cell lines (Islam *et al.* 1992, 1998; Gromada *et al.* 1995; Gamberucci *et al.* 1999; Maechler *et al.* 1999; Holz *et al.* 1999; Kang *et al.* 2001, 2003; Kang & Holz, 2003; Mitchell *et al.* 2003; Sasaki *et al.* 2002; Varadi & Rutter, 2002; Bruton *et al.* 2003; Tsuboi *et al.* 2003; but see Dyachok & Gylfe, 2004). The source of Ca^2+^ mobilized may include the ER because we find that caffeine and forskolin fail to promote CICR in thapsigargin-treated cells. Moreover, caffeine releases ER Ca^2+^, as measured in INS-1 cells expressing YC3.3-er.

RYR is implicated in the regulation of [Ca^2+^]_i_, exocytosis, apoptosis, and endosome function in β cells (Islam *et al.* 1992, 1998; Takasawa *et al.* 1998; Holz *et al.* 1999; Lemmens *et al.* 2001; Quesada *et al.* 2002; Bruton *et al.* 2003; Kang & Holz, 2003; Johnson *et al.* 2004*a*,*b*). Because not all studies support these contentions (Tengholm *et al.* 1998, 1999, 2000), there may exist differences in the levels of expression of RYR when comparing strains of mice or when making comparisons across species lines. The failure of prior studies to detect RYR in β cells might also be explained by the use of an experimental strategy that relies on treatment of cells with diazoxide and verapamil (Dyachok & Gylfe, 2004; Dyachok *et al.* 2004). These agents disrupt CICR by virtue of their ability to inhibit Ca^2+^ influx (Meissner, 2002). In contrast, human β cells not treated with these agents exhibit an increase of [Ca^2+^]_i_ in response to a low concentration of ryanodine, thereby demonstrating the presence of RYR in this cell type (Johnson *et al.* 2004*a*). Similarly, human β cells not treated with diazoxide or verapamil exhibit an increase of [Ca^2+^]_i_ in response to GLP-1, an action inhibited by a high concentration of ryanodine (Holz *et al.* 1999).

New studies demonstrate the presence of immunologically detectable RYR in human and mouse β cells (Johnson *et al.* 2004*a*,*b*). Although the molecular nature of β cell RYR remains to be determined, it may exhibit features somewhat different from the isoforms described to date. Indeed, splice variants of *RYR1* and *RYR2* mRNA are expressed in islets (Lee *et al.* 2002; Okamoto *et al.* 2004). Sequence variations of this type may explain why some have found it difficult or impossible to detect *RYR* mRNA by RT-PCR of purified preparations of mouse β cells (Beauvois *et al.* 2004).

### Potential IP_3_-R-mediated signalling properties of the GLP-1-R

Actions of GLP-1 mediated by the IP_3_-R are also of interest. Although activation of the GLP-1-R fails to stimulate IP_3_ production in islets or INS-1 cells (Fridolf & Ahren, 1991; Zawalich *et al.* 1993; Kang *et al.* 2003), cAMP can, under certain conditions, facilitate IP_3_-R function. This action of cAMP is PKA mediated (Bruce *et al.* 2003), and is reported to be operational in mouse β cells (Liu *et al.* 1996; Dyachok *et al.* 2004). Mobilization of Ca^2+^ from IP_3_-R-regulated Ca^2+^ stores may explain, at least in part, how GLP-1 stimulates exocytosis in β cells (Dyachok *et al.* 2004). It may also play some role in the stimulation of β cell mitochondrial ATP production (Tsuboi *et al.* 2003) and mitogen-activated protein (MAP) kinase signalling (Arnette *et al.* 2003). Therefore, it is noteworthy that we demonstrate IP_3_-R-mediated CICR sensitized by a cAMP-elevating agent and which is blocked by treatment of β cells with heparin, an inhibitor of IP_3_-R function.

It is also important to note that Ca^2+^ exerts stimulatory effects on IP_3_ production in insulin-secreting cells (Roe *et al.* 1993; Gromada *et al.* 1996). This action of Ca^2+^ is most likely a consequence of its ability to activate phospholipase C (Berridge *et al.* 2003). If such an action of Ca^2+^ were to exist in β cells loaded with NP-EGTA, the uncaging of Ca^2+^ might raise levels of IP_3_, thereby stimulating the IP_3_-R. CICR triggered in this manner might be facilitated under conditions in which the IP_3_-R is sensitized by cAMP-elevating agents. Because evidence also exists for ‘atypical’ mechanisms of Ca^2+^ release in the β cell (Johnson & Misler, 2002; Masgrau *et al.* 2003; Mitchell *et al.* 2003; Beauvois *et al.* 2004), actions of cAMP-elevating agents described here may not be restricted to RYR or the IP_3_-R.

### Epac-mediated sensitization of CICR

Prior studies of multiple cell types demonstrate that cAMP acts via PKA to sensitize RYR and the IP_3_-R (Marx *et al.* 2000; Bruce *et al.* 2003). Here we report that the action of cAMP may also be Epac-mediated. A cAMP analogue selective for Epac sensitizes the CICR mechanism of β cells, whereas transfection of INS-1 cells with dominant negative Epac diminishes CICR. Although prior studies implicate Epac2 in this process (Kang *et al.* 2001, 2003; Tsuboi *et al.* 2003), we are the first to demonstrate that Epac1 may also be a contributing factor. One established downstream effector of Epac is the small-molecular weight GTPase Rap. Although the signalling properties of Rap are not fully understood, evidence exists that it promotes protein kinase-mediated phosphorylation independently of PKA (Holz, 2004*a*). Therefore, cAMP-dependent sensitization of intracellular Ca^2+^ release channels might require formation of an activated Epac/Rap signalling complex. Such an action of Epac would complement its ability to interact with secretory granule-associated proteins (Kashima *et al.* 2001; Shibasaki *et al.* 2004) and to regulate fast Ca^2+^-dependent exocytosis in the β cell (Eliasson *et al.* 2003).

### Conclusion

Findings presented here suggest that RYR and IP_3_-R Ca^2+^ release channels mediate the Ca^2+^ mobilizing action of GLP-1 in β cells. Both types of Ca^2+^ release channels act as cAMP and Ca^2+^ coincidence detectors, because their opening is facilitated by a simultaneous increase of intracellular cAMP and Ca^2+^ concentrations. PKA and Epac-mediated sensitization of CICR demonstrated here may provide a new explanation for how GLP-1 interacts with β cell glucose metabolism to stimulate insulin secretion.

## Supplemental material

The online version of this paper can be accessed at: DOI: 10.1113/jphysiol.2005.087510

http://jp.physoc.org/cgi/content/full/jphysiol.2005.087510/DC1 and contains supplemental material consisting of four figures.

This material can also be found as part of the full-text HTML version available from http://www.blackwell-synergy.com
