# Supplementary material for: A cAMP and Ca2+ coincidence detector in support of Ca2+-induced Ca2+ release in mouse pancreatic β cells
Source: J Physiol. 2005 Apr 28;566(1):173–88. doi: 10.1113/jphysiol.2005.087510 (PMC3583090; doi:10.1113/jphysiol.2005.087510)

Online Supplementary Material

A cAMP and Ca^{2+} coincidence detector in support of Ca^{2+}-induced Ca^{2+} release (CICR) in pancreatic β cells

Guoxin Kang[1], Oleg G. Chepurny[1], Michael J. Rindler[2], Leon Collis[3], Zina Chepurny[1], Wen-hong Li[4], Mark Harbeck[5], Michael W. Roe[5], and George G. Holz[1]*

Department of Physiology and Neuroscience[1]
Department of Cell Biology[2]
Department of Cardiology[3]
New York University School of Medicine
New York, NY 10016 USA

Departments of Cell Biology and of Biochemistry[4]
University of Texas Southwestern Medical Center
5323 Harry Hines Blvd.
Dallas, TX 75390-9039

Department of Medicine[4]
MC1027, AMB M172
The University of Chicago
5841 South Maryland Avenue
Chicago, IL 60637

*Address Correspondence To:

George G. Holz, Ph.D.
Associate Professor of Physiology and Neuroscience
Medical Sciences Building Room 442
550 First Avenue, New York, NY 10016
Tel 212-263-5434
Fax 212-689-9060
Email: holzg01@popmail.med.nyu.edu

Supplementary Material

Figure 1. Optical components of the UV flash photolysis and digital imaging systems used to measure EYFP and fura-2 fluorescence in cells loaded with NP-EGTA. Abbreviations: Ex., excitation wavelength; Em, emission wavelength; LP, long pass filter; SP, short pass filter; DM, dichroic mirror; ICCD, intensified charge coupled device; CMV, cytomegalovirus promoter; EYFP, enhanced yellow fluorescent protein.

Figure 2. EYFP was not detected using a fura-2 filter set. ***A1, left panel***: an INS-1 cell expressing EYFP imaged using an EYFP filter set and a camera gain of 1X. This cell was not loaded with fura-2. ***A1, middle panel***: the same cell imaged using a fura-2 filter set (Ex. 380 nm; Em. 510 nm) with the camera gain set to 10X. ***A1, right panel***: the same cell imaged using a fura-2 filter set (Ex. 340 nm; Em. 510 nm) with the camera gain set to 10X. Note that no EYFP fluorescence was measurable when using the fura-2 filter sets. For all experiments described in the main body of the manuscript, the camera gain was set to 1X when performing measurements of EYFP. ***A2,*** the fluorescence intensities (arbitrary light units) measured within the zones defined by the white rectangles in ***A1***. ***A3,*** the average fluorescence intensities (***n*** = 6 cells per histogram bar) measured using the EYFP or fura-2 filter sets in cells expressing EYFP (+) or in control cells not expressing EYFP (-). No fura-2 was present for the analyses illustrated in ***A1-A3***. Scale bars corresponds to 6 microns.

Figure 3. Fura-2 was not detected using an EYFP filter set. ***A1, left panel***: an INS-1 cell loaded with fura-2 and imaged using an EYFP filter set (Ex. 513 nm; Em. 527 nm) and a camera gain of 10X. This cell was not expressing EYFP. ***A1, middle panel***: the same cell imaged using a fura-2 filter set (Ex. 380 nm; Em 510 nm) with the camera gain set to 10X. ***A1, right panel***: the same cell imaged using a fura-2 filter set (Ex. 340 nm; Em. 510 nm) with the camera gain set to 10X. Note that no fura-2 fluorescence was measurable when using the EYFP filter set. For all experiments described in the main body of the manuscript, the camera gain was set to 10X when performing measurements of fura-2. ***A2,*** the fluorescence intensities (arbitrary light units) measured within the zones defined by the white rectangles in ***A1***. ***A3,*** the average fluorescence intensities (***n*** = 5 cells per histogram bar) measured using the EYFP or fura-2 filter sets in cells loaded with fura-2 (+) or in control cells not loaded with fura-2 (-). No EYFP was present for analyses illustrated ***A1-A3***. Scale bars corresponds to 6 microns.

Figure 4. Even at a camera gain of 100X, EYFP was barely detectable when using a fura-2 filter set. ***A1,*** an INS-1 cell expressing EYFP and imaged using an EYFP filter set and a camera gain of 1X. This cell was not loaded with fura-2. ***B,*** the same cell imaged using a fura-2 filter set and a camera gain of 10X. ***C,*** the same cell imaged using a fura-2 filter set and a camera gain of 100X. No fura-2 was present for the analyses illustrated in ***A-C***. Scale bars corresponds to 6 microns.

Supplementary Fig.1 Kang et al.

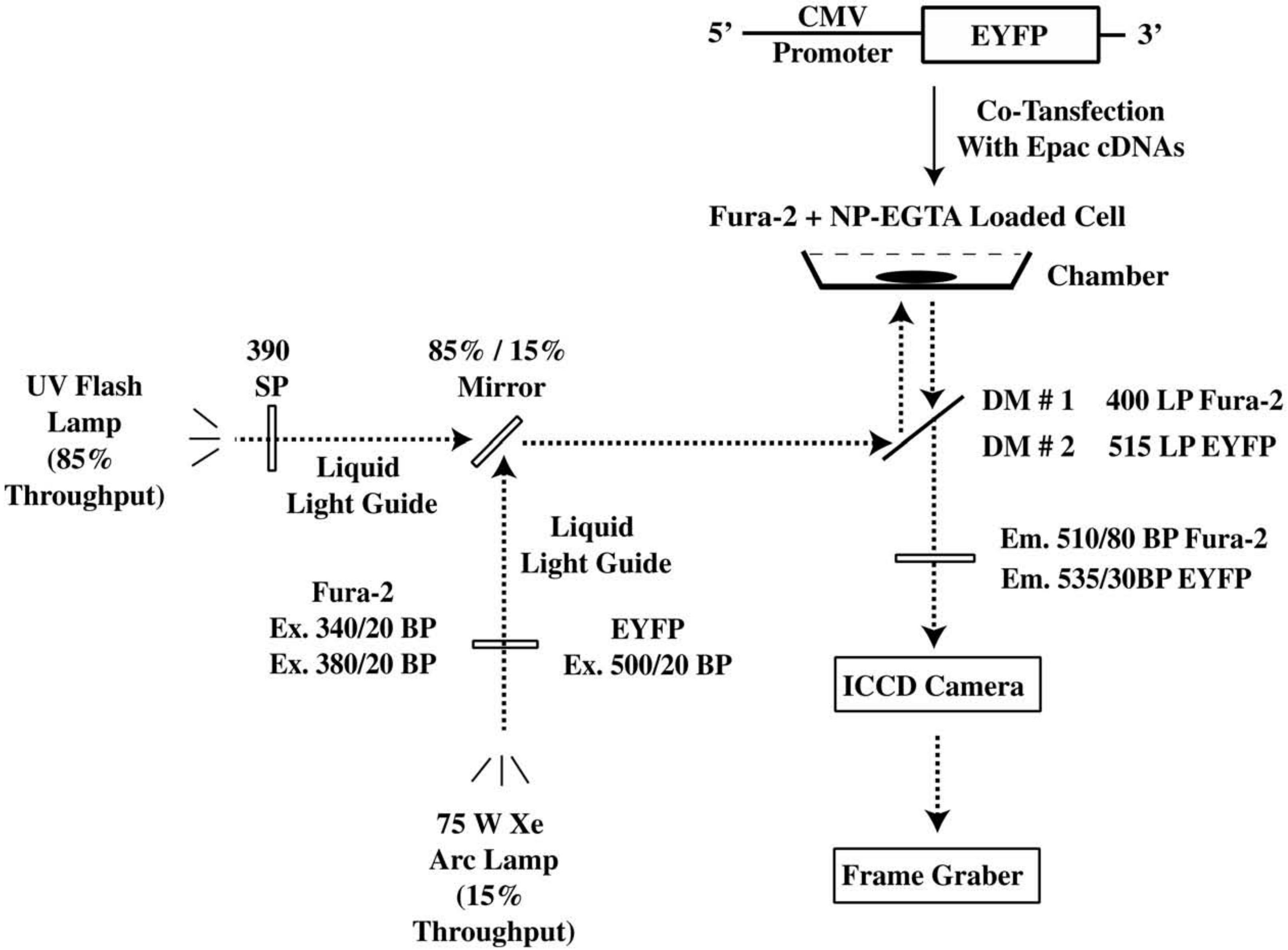

Supplementary Fig.2 Kang et al.

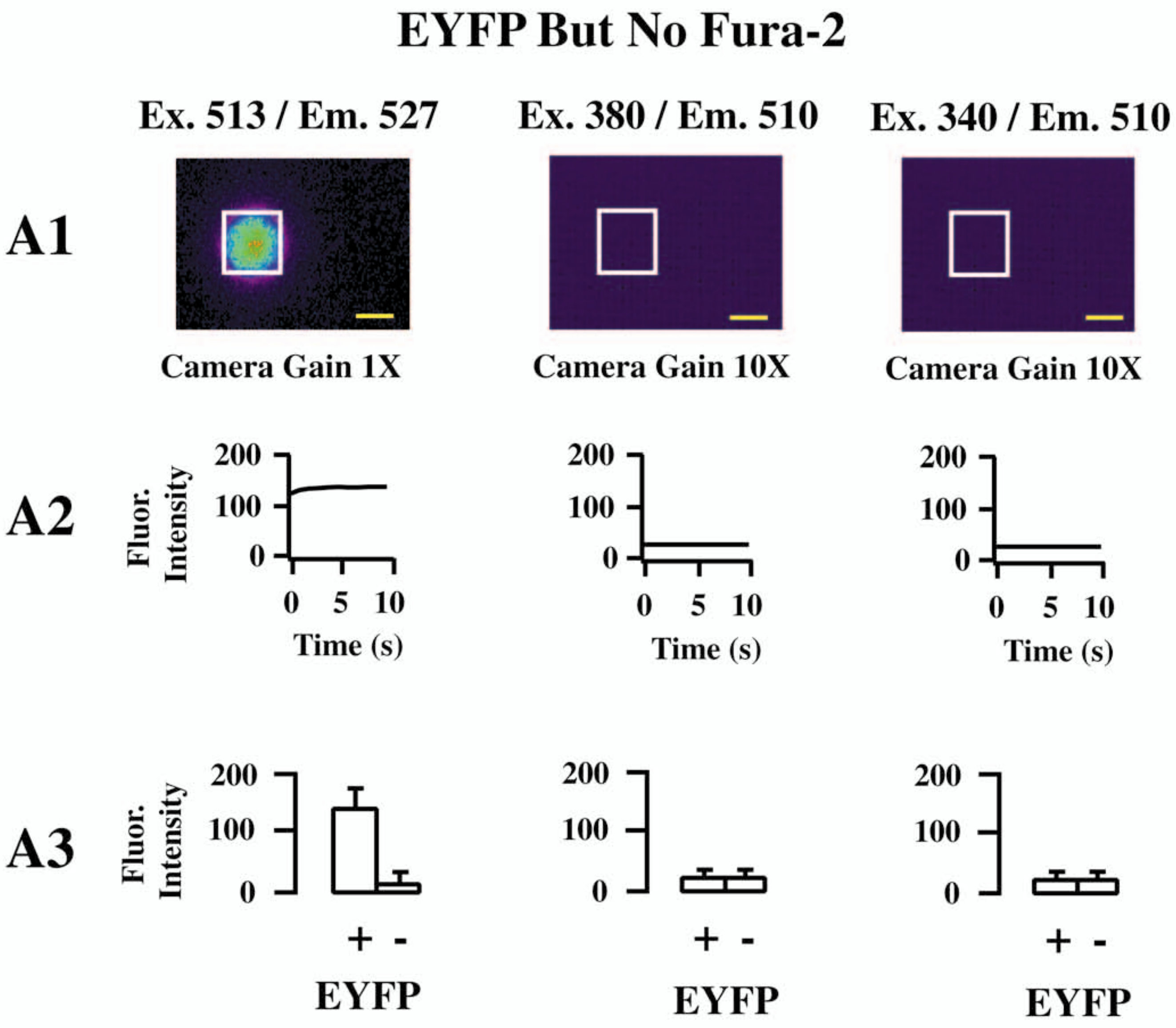

Supplementary Fig.3 Kang et al.

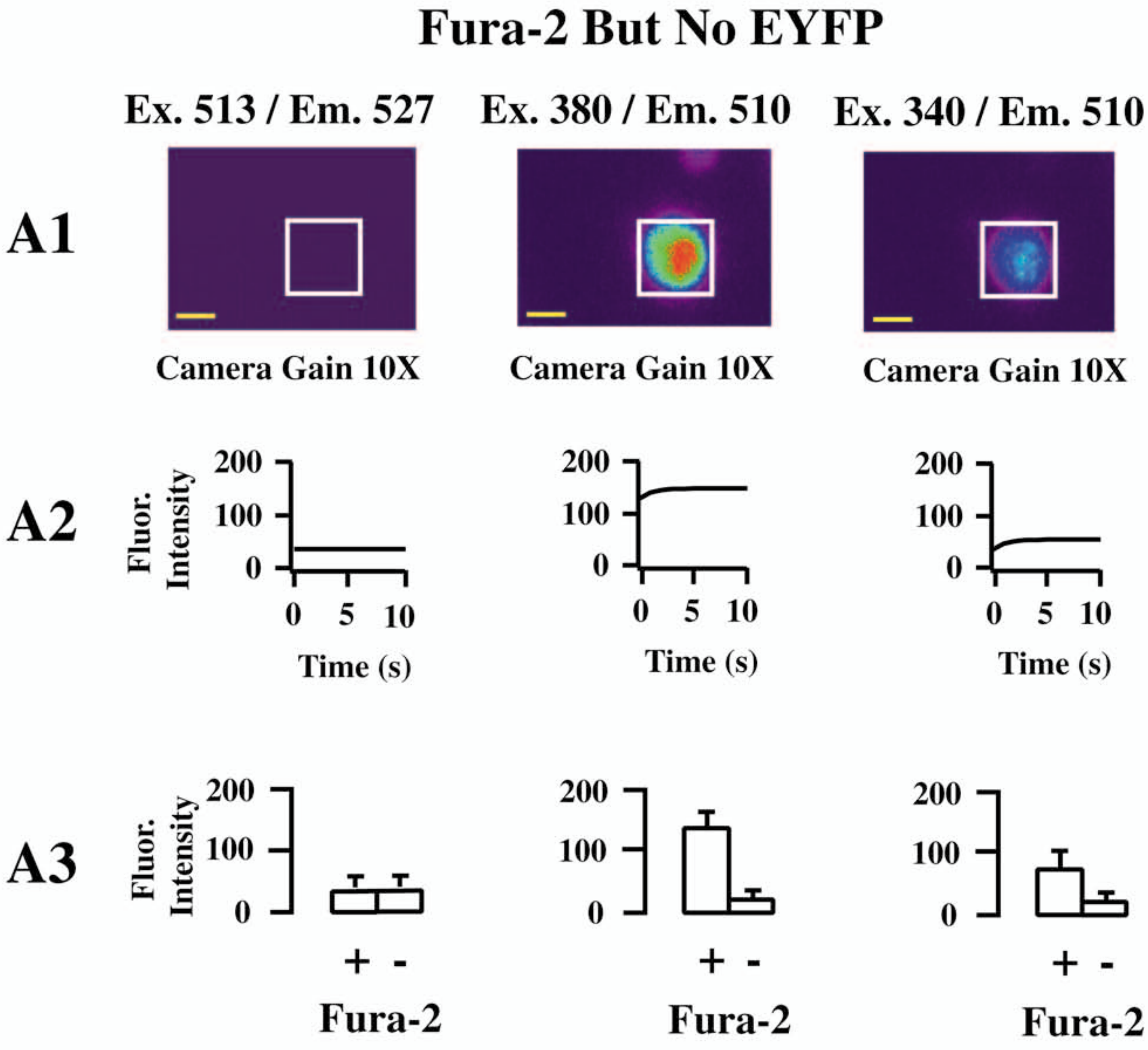

Supplementary Fig.4 Kang et al.

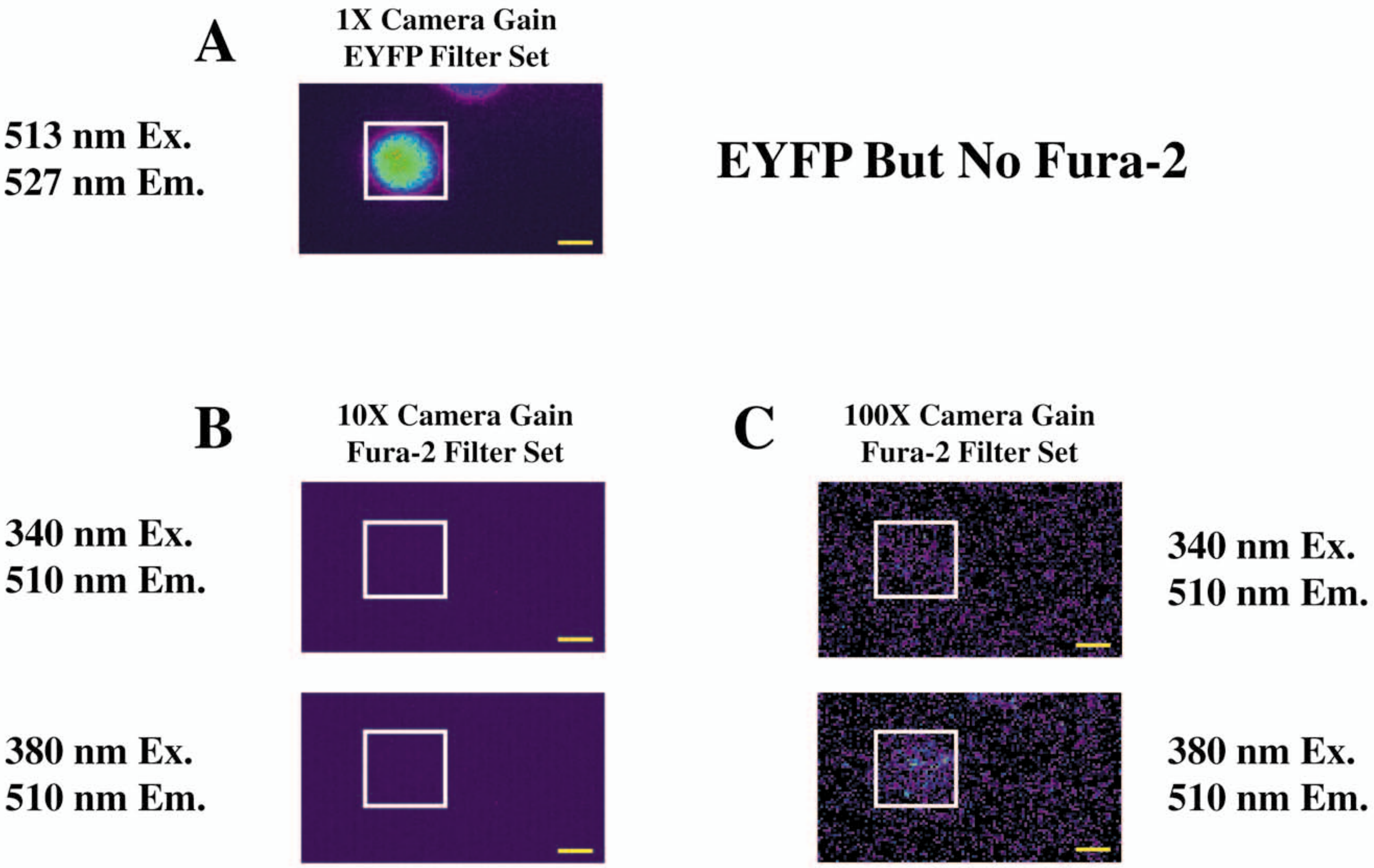

Supplement: Supplementary file 1 [file tjp0566-0173-sd1.pdf]
